# Virucidal Efficacy of Laser-Generated Copper Nanoparticle Coatings against Model Coronavirus and Herpesvirus

**DOI:** 10.1021/acsami.5c03330

**Published:** 2025-04-22

**Authors:** Shahd Bakhet, Rasa Mardosaitė, Mohamed Ahmed Baba, Asta Tamulevičienė, Brigita Abakevičienė, Tomas Klinavičius, Kristupas Dagilis, Simas Račkauskas, Sigitas Tamulevičius, Raimundas Lelešius, Dainius Zienius, Algirdas Šalomskas, Krišja̅nis Šmits, Tomas Tamulevičius

**Affiliations:** † Institute of Materials Science of Kaunas University of Technology, K. Baršausko Street 59, LT-51423 Kaunas, Lithuania; ‡ Department of Physics, Kaunas University of Technology, Studentų Street 50, LT-51368 Kaunas, Lithuania; § Department of Veterinary Pathobiology, Lithuanian University of Health Sciences, Tilžės Street 18, LT-47181 Kaunas, Lithuania; ∥ Institute of Microbiology and Virology, Lithuanian University of Health Sciences, Tilžės Street 18, LT-47181 Kaunas, Lithuania; ⊥ Institute of Solid State Physics, University of Latvia, 8 Kengaraga Street, LV-1063 Riga, Latvia

**Keywords:** laser ablation in liquid, copper nanoparticles, spray-coating, virucidal surface, coronavirus, herpesvirus

## Abstract

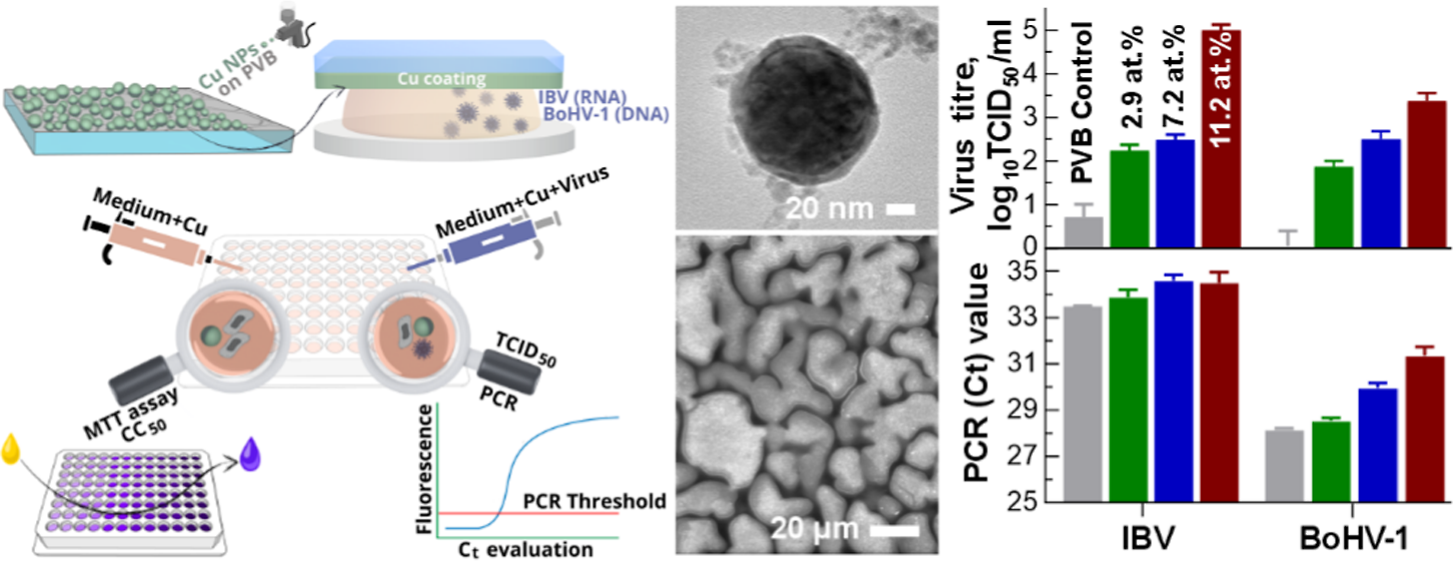

High-efficiency antiviral surfaces can be an effective means of fighting viral diseases, such as the recent COVID-19 pandemic. Copper and copper oxides, their nanoparticles (NPs) (CuNPs), and coatings are among the effective antiviral materials having internal and external biocidal effects on viruses. In this work, CuNP colloids were produced via femtosecond laser ablation of the metal target in water, a photophysical, cost-effective green synthesis alternative utilizing sodium citrate surfactant stabilizing the NPs. Raman spectroscopy and X-ray diffraction studies confirmed that the 32 nm mean size CuNPs are mixtures of mainly metallic copper and copper(I) oxide. Polyvinyl butyral was utilized as the binding agent for the CuNPs deposited via high-throughput spray-coating technology. The virucidal efficacy of such coatings containing Cu content ranging from 2.9 to 11.2 atom % was confirmed against animal-origin coronavirus containing ribonucleic acid, the agent of avian infectious bronchitis (IBV), and herpesvirus containing DNA, the agent of bovine herpesvirus (BoHV-1) infection. It was demonstrated that after a short time of exposure, the Cu NP-based coatings do not have a toxic effect on the cell cultures while demonstrating a negative effect on the biological activity of both model viruses that was confirmed by quantification of the viruses via the determination of tissue culture infectious dose (TCID_50_) virus titer and their viral nucleic acids via determination of threshold cycle (Ct) employing real-time polymerase chain reaction analysis. The assays showed that the decrease in TCID_50_ virus titer and increase in Ct values correlated with Cu content in Cu NP-based coatings for both investigated viruses. Contact with coatings decreased IBV and BoHV-1 numbers from 99.42% to 100.00% and from 98.65% to 99.96%, respectively. These findings suggest that CuNPs show inhibitory effects leading to the inactivation of viruses and their nuclei regardless of the presence of a viral envelope.

## 1 Introduction

Respiratory infections caused by different pathogens affect more people than any other infection around the world. In the last years according to the World Health Organization, close to 800 million people had COVID-19 and more than 7 million related deaths were counted in the year 2024.^1^ Having in mind the overall population exceeding 8 billion,^2^ one can estimate that over 8% of people suffered from the disease and statistically more than one vaccine was applied per person as over 13 billion vaccine doses were administered. Although COVID-19 is no longer a pandemic, depending on the season, more than 20,000 cases are registered daily around the world. One of the ways to deal with the outbreaks is good hygiene, which can be implemented by using antiviral surfaces effective against ribonucleic acid (RNA) like viruses such as coronavirus and influenza virus.^3^ The studies showed that there are significant differences in the biophysical stability factors of these viruses on the surfaces of different materials (plastic, stainless steel, cardboard, and copper), and this led the researchers to investigate different hypotheses to evaluate the physical and chemical properties of the surfaces of various coatings (including on face masks) for possibly reducing the stability of SARS-CoV-2 and thus its potential transmission to healthy people. Several key properties that affect the survival of viruses on surfaces were identified.^4^ The first one is the contact material’s shape and type of surface, such as porosity and roughness down to the nanoscale dimensions. The second is physical abiotic factors that affect the surface of the material, such as relative humidity, temperature, and exposure to light (rays of different solar spectra). The third is chemical, such as pH, the presence of reactive ions, the state of adsorption, and the presence of organic matter or specific chemical components in the environment. It should be emphasized that many viruses, depending on their external biochemical structure, can be stabilized and protected by surrounding organic matter, such as saliva or mucus droplets. In this case, the presence of substances and external structures such as bacteria biofilms, fats, proteins, or carbohydrates in the contact environment of the virus can additionally and significantly increase their structural stability and resistance.^4^

Different combinations of metal oxide materials concerning biological activity were studied in various biosystems, but silver and copper derivatives and their compounds were among the most effective ones, even though the means of their internal and external virucidal activity mechanisms against viruses are still not fully understood. At the same time, metal ions are an integral part of some viral proteins and play an important role in their survival and pathogenesis.^5^ Cu is an indispensable trace element for a wide range of biological functions in all living systems, and due to its potent antiviral and antioxidant properties, it may be promising even for prophylactic modalities against COVID-19 infection.^6^ In recent years, there has been increasing interest in the use of self-disinfecting surfaces to reduce the concentration of different pathogens to prevent the spread of diseases. Various materials were used as additional safety measures, for example, surface coatings with ZnO^7^ and CuO nanoparticles (NPs).^8^ Due to its effective contact denaturing effect and price, Cu is the most used metal for the development of antimicrobial surfaces these days. It can be used pure, alloyed, in composites with different structures, and NPs can be used in a variety of products such as door handles, railings, and textiles.^9^ Various forms of Cu compounds, especially copper oxide NPs (CuO NPs), are some of the most popular in the field of antimicrobial research. These NPs have been widely adopted in the industry, due to their versatile physical properties and low cost of production.^10^ Cu NPs with graphene had a potent inactivation effect on viruses and when combined with other metals increased their antiviral properties,^3^ or had an expressed effect on the virus’s half-life,^11^ but most studies so far have focused on the effectiveness of CuO against bacteria.^10^ The effect of Cu NPs on viruses is still in its early stages of investigation, which can correspond to understanding the virus inhibition mechanism.^12^

Cu and CuO NPs can be synthesized by exploiting various approaches, but the photophysical method utilizing ultrashort laser sources has attracted a lot of attention. The advent of high peak power ultrashort lasers and the scalability of the laser ablation process offer grams per hour reaching yields that already surpass the process cost of traditional chemical synthesis, even at the industrial synthesis level.^13^ Laser ablation offers distinct advantages over conventional material shredding techniques, particularly in the synthesis of NPs. Virtually any solid material can be transformed into an NP colloid employing laser ablation of the target in liquid.^14^ A liquid lowers the heat load on the target, confines the vapor and plasma, and increases the shock pressure on the surface. The ablated material disperses into the liquid and nucleates into the NPs. The fraction of the ablated material is inevitably chemically oxidized or reduced on the surface of the NPs.^15^ NP oxidation in water is a straightforward and effective method for producing oxide and metal core-oxide shell nanomaterials.^16^ Cu and its derivative NPs can also be produced by reductive laser ablation of Cu precursor suspensions^17^ or by laser fragmentation of micrometer-sized powder in liquid.^17,18^

The physical properties of the used solvent play a crucial role in determining the stability, structure, and crystallinity of laser-ablated Cu NPs.^16,19,20^ For example, when made in pure water, Cu NPs were least stable, while the aging of those synthesized in ethanol and acetone was slower, as seen from the plasmon peak-related color changes.^21^ Many different solvents in addition to water have been investigated, including ethanol,^21^ acetone,^16,19^ decane,^22^ and so on. Under laser ablation of the target in liquid, Cu mainly forms two stable oxides: monoclinic cupric oxide CuO and cubic cuprous oxide Cu_2_O.^23^ Metastable paramelaconite Cu_4_O_3_ particles were also reported in Cu ablation experiments.^22^ Gondal et al. reported two copper phases Cu/Cu_2_O in a water solution that were changed into Cu/CuO when a hydrogen peroxide oxidizing agent was present in the liquid medium.^24^ Nath et al. demonstrated that under tightly focused ablation conditions, CuO formation with typical 200 nm diameters was observed. In contrast, under defocusing conditions, Cu_2_O nanocolloids of particles smaller than 10 nm were obtained.^23^ Aging of Cu derivatives in a natural environment^21^ can be mitigated by applying a capping layer on NPs or using a stabilizing matrix, for example, copper-graphene nanocomposite,^3^ Cu_2_O NPs bound with polyurethane,^25^ Cu_2_O nanocolloid using sodium alginate as a capping agent;^26^ Cu NPs in polyaniline,^27^ Cu-NP-containing shellac biopolymer resin,^28^ Cu NPs in polylactic acid,^29^ and Cu nanowires in Zeolitic imidazolate framework.

The reported antiviral and antibacterial efficacy of liquid-suspended Cu and copper iodide^30^ NPs appears promising, but in real-life situations, copper-based antiviral coatings find more practical applications. A variety of effective Cu film and coating deposition methods indicated well-expressed antiviral activity, including simple drop-casting of Cu colloid on glass substrates,^31^ Cu composite polymer deposition using a sponge,^25^ application of Cu-containing paint,^32^ dip-coating and sol–gel synthesis,^33^ self-assembly of Cu-binding peptide,^34^ thermal evaporations of the Cu thin film and annealing,^35^ magnetron sputtering of the Cu thin film after ion beam treatment,^36^ reactive magnetron sputtering of Cu/CuO nanocomposite films amorphous carbon matrix,^37^ flame deposition of Cu oxide NPs,^38^ cold spraying of Cu particles with a high-pressure carrier gas,^39^ and dealloying the Mn–40Cu alloy surface.^40^ The spray deposition alternative is advantageous in a way that the Cu NP coating can be easily applied both on flat^41^ and on elaborated surfaces like textiles;^42^ also, it can be deposited layer by layer^43^ forming a functional surface and the method is easily scalable as it is varied out at the atmospheric conditions.

This work presents a cost-effective method to produce efficient antiviral Cu-NP-based coatings produced by high-throughput spray-coating. The femtosecond laser ablation of the Cu target in water with sodium citrate was used to generate chemically stable colloidal Cu NPs, which were applied as active materials for developing antiviral surfaces. Polyvinyl butyral was utilized as the binding agent for the spray-coated Cu NPs. The virucidal efficacy of coatings containing different Cu NP contents was confirmed against animal-derived model viruses, namely, RNA-containing coronavirus and deoxyribonucleic acid (DNA) containing herpesvirus.

## 2 Methods

### 2.1 Production and Stabilization of Copper Nanoparticles

**Figure 1:**
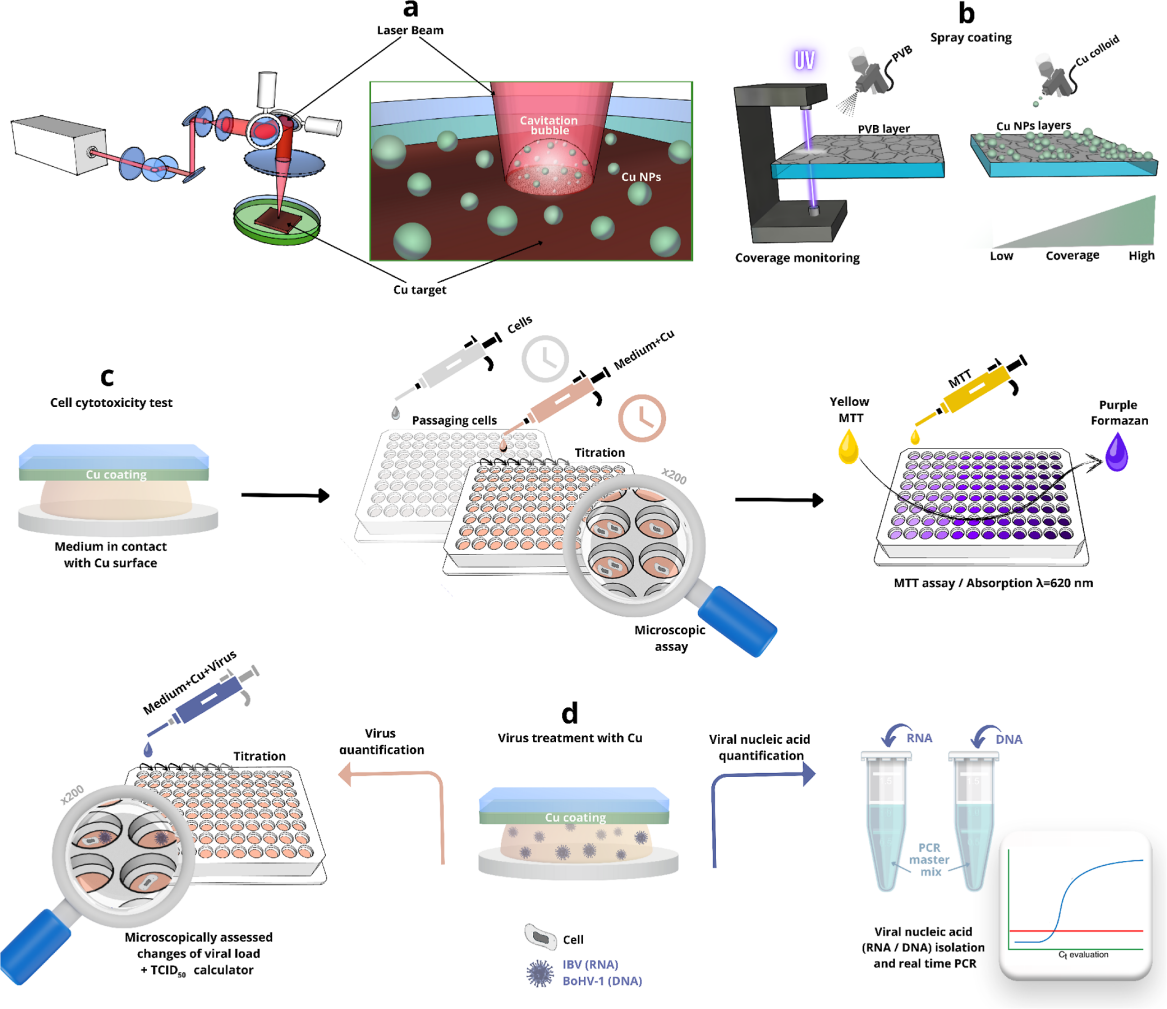
Preparation of Cu NP-loaded coatings and general scheme of the in vitro experiments. (a) Femtosecond laser micromachining setup with a galvanometric scanner and close-up view of the processes in the Petri dish where the Cu target in liquid is scanned with a laser during the ablation process; (b) two-stage spray-coating process starting with deposition of PVB, which is followed by deposition of Cu-NPs, the UV light transmission meter is used for monitoring effective thicknesses of the coatings; (c) cell cytotoxicity test; (d) treatment of virus culture by contacting with investigated coatings and preparation of samples for two quantitative analyses, i.e., virus quantification employing calculating tissue culture infectious dose, and viral nucleic acids via real-time polymerase chain reaction.

Colloidal Cu NPs were produced by laser ablation of a pure copper (99.9%) metal target (acquired from the Lithuanian Mint, Lithuania) in ultrapure type 1 water (18.2 MΩ/cm) with varying concentrations of sodium citrate surfactant from 0.002 to 2 mmol (Sigma-Aldrich, USA). A 0.02 mmol concentration was selected for further experiments based on the stability study described in the Supporting Information. The metal target was sonicated in acetone to eliminate any organic contamination before the ablation. The target was placed in a glass beaker and filled with 14 mL of an aqueous solution, which ensured approximately 4 mm of the liquid layer above the flat surface of the metal target. The laser ablation was carried out employing a laser micromachining workstation (FemtoLab, Altechna R&D, Lithuania) and a 270 fs pulse length, 1030 nm wavelength Yb:KGW femtosecond laser (Pharos, Light Conversion, Lithuania) operating at 200 kHz repetition rate and 3 W average power. The laser beam was scanned with a galvanometer scanner (SCANcube III 14, ScanLab, Germany) and focused down to ca. 20 μm spot size by a telecentric 163 mm focal distance f-Theta lens (SillOptics, Germany). The focal plane with respect to the metal target surface was adjusted with a motorized *z* stage (Physik Instrumente, Germany). More details about the used setup are described elsewhere.^44^ The sample treatment was controlled with the SCA software (Altechna R&D, Lithuania) and the surface area of 3 mm × 3 mm was scanned by 30 μm separated lines at a 20 mm/s speed, repeating the process 300 times. The overall treatment for each batch of colloid lasted ca. 79 min. The principal scheme of the laser ablation setup and process is depicted in Figure 1a. The described process was repeated several times, and the colloid was exchanged with water. The resulting overall colloid volume was concentrated, and the solvent was exchanged with isopropanol by employing centrifugation as described in the Supporting Information and depicted in Figure S1. The resulting Cu NP colloid was used for the spray coating.

### 2.2 Spray Coating

The deposition of the investigated films on the 1 cm × 1 cm soda lime glass microscope slide substrates (Chemland, Poland) was carried out in two steps. After cleaning the substrates in ethanol (99.5%, Emparta, Merk, Germany), they were first spray-coated with poly(vinyl butyral) of 3 mg/mL concentration in isopropanol (99.7%, Chempur, Poland). PVB spray coating was carried out with an aerograph AD-776B Airbrush MAR EW-6000B (Adler, Poland) employing a 0.2 mm nozzle (air pressure 2 bar) from a constant 10 cm distance. The effective thickness of the layer was monitored with the custom-made UV light transmission meter described elsewhere^45^ and kept at a 3% optical extinction level throughout the samples. The spray-coating process is summarized in Figure 1b. Several PVB-coated glass samples, termed “PVB”, were used as prepared for antiviral testing as a control. Then, in the second step, the Cu NP colloid ink was spray-coated on the PVB layer at 1 bar of pressure. The concentration of deposited Cu was controlled by varying the duration of deposition and was also monitored with the UV transmission meter, indicating optical extinction from 10% to 25%, which correlated with the Cu NP content.^45^ The samples were termed “PVB + CuO 10%”, “PVB + CuO 15%”, and “PVB + CuO 25%”.

### 2.3 Physical Characterization of the NPs and the Coating

The optical density of the resulting Cu colloids and their stability over time were measured in the UV–vis–NIR spectral range employing AvaSpec-2048 (Avantes, The Netherlands) fiber spectrometer of 1.4 nm spectral resolution and a combined deuterium and halogen light source AvaLight-DHc (Avantes, The Netherlands).

The Cu NP size distribution was evaluated from field emission gun scanning electron microscope (SEM) Quanta 200 FEG (FEI, Czech Republic) micrographs. The diameters of the NPs were extracted from SEM images using a custom multistep processing algorithm implemented in MATLAB (MathWorks, USA). Image binarization (separation of particles from their background) was performed using adaptive thresholding. Stuck-together particles were segmented using the watershed transformation, similar to Baiyasi et al.^46^ The diameter of each particle was determined from the occupied area, assuming that particles are round.

The structure of the NPs was further investigated with a transmission electron microscope Tecnai G2 F20 X-TWIN (FEI, Czech Republic). Selected area electron diffraction (SAED) results were analyzed using CrysTBox software.^47^ The Cu content in the coatings was obtained with the energy-dispersive X-ray spectrometer (EDS) Quantax 200 (Bruker, Germany) attached to SEM under a 5 kV accelerating voltage.

The crystal structure of Cu NPs was determined using a D8 Discover X-ray diffractometer (XRD, Bruker AXS GmbH, Germany) operating at 40 kV and 40 mA with a Cu K_α_ (λ = 1.5418 Å) radiation source and parallel beam geometry with a 60 mm Göbel mirror. Soller slit with an axial divergence of 2.5° was utilized on the primary side. Diffraction patterns were recorded using a fast-counting LynxEye detector with a 2.475° opening angle and 6 mm slit opening. The peak intensities were scanned over the range of 20 – 90° (coupled 2θ–θ scans) with 0.02° step size.

Raman scattering measurements of Cu NPs were carried out employing an InVia (Renishaw, UK) spectrometer and a 532 nm laser focused with 50× objective 0.75NA lens. For the analysis, a 50 μL drop of Cu colloidal solution was dried on a glass substrate.

The PVB with Cu NP coatings on glass substrate hardness measurements were tested by applying 1, 2, and 3 N force using a sclerometer Elcometer 3092, according to standard AS 3894.4 (EN 438-2, ISO 4586-2). The impact was investigated under an optical microscope. More details are provided in the Supporting Information.

### 2.4 Antiviral Testing

#### 2.4.1 Model Viral Strains and Cell Cultivation

The developed coatings were tested with two types of viruses: (i) animal-origin coronavirus containing RNA, the agent of avian infectious bronchitis (IBV), and (ii) herpesvirus containing DNA, the agent of bovine herpesvirus infection (BoHV-1). Viruses were propagated using two kinds of cell cultures, i.e., Vero and MDBK/NBL-1, respectively.

The Vero-adapted cytopathogenic IBV Beaudette strain was obtained from Dr M. H. Verheije (Utrecht University, The Netherlands). Madin–Darby bovine kidney (MDBK)-adapted, cytopathogenic BoHV-1 4016 strain^48^ was provided by Dr I. Jacevičienė (National Food and Veterinary Risk Assessment Institute, Lithuania). The viruses were stored in a deep freezer at −80 °C.

Vero (ATCC CCL-81) and MDBK (NBL-1, Bov.90050801) cell cultures were obtained from Dr I. Jacevičiene(National Food and Veterinary Risk Assessment Institute, Lithuania). Vero and MDBK/NBL-1 cells were cultured in Dulbecco’s Modified Eagle’s Medium (DMEM, Gibco, UK) and DMEM/Nutrient Mixture F-12 (DMEM/F-12, Gibco, UK), respectively, with 10% fetal bovine serum (FBS, Gibco, UK) at 37 °C in a 5% CO_2_ incubator. Nystatin (100 units/ml) and Gentamicin (50 μg/mL) were used to prevent microbial contamination for Vero cells, while Penicillin (100 units/ml) and Streptomycin (100 μg/mL) were used to prevent microbial contamination for MDBK/NBL-1 cells. Depending on the description of the method, the tests were performed at temperatures of 20 ± 2 °C (class II biosafety cabinet) and 37 °C (cell incubation).

#### 2.4.2 Cytotoxicity Control

The cytotoxicity of different Cu content-containing coatings was determined in Vero and MDBK cells using the MTT assay.^49^ First, cells were passaged at a concentration of 1 × 10^4^ cells/well in a 96-well plate (TPP, Switzerland) and grown at 37 °C for 24 h. Then 10 μL of medium containing 2% FBS was placed on the surface of the 6 well tissue culture test plate (TPP, Switzerland), covered with the surface of the 1 cm × 1 cm sized glass substrates with the investigated coatings, and incubated at room temperature (21 ± 1 °C) for 1 h. The thickness of the medium layer was ca. 100 μm. After incubation, the liquids were initially diluted 1:50 with a medium containing 2% FBS, and then, employing 2-fold dilution steps, the volumes of 100 μL were prepared for the treatment of cells. Dilutions from 1:50 to 1:1600 were prepared for the evaluation of cytotoxicity.

Later, the growth medium was removed from the cell culture plate wells, and cells were washed twice with phosphate-buffered saline, and treated with 100 μL of titrated liquid at 37 °C in a 5% CO_2_ incubator for 72 h. Before the MTT assay, the cells were microscopically observed and compared with control cells. After 72 h, MTT reagent (10 μL, 5 mg/mL, Sigma-Aldrich) was added and incubated for 4 h at 37 °C. Then the liquid was carefully discarded and 100 μL dimethyl sulfoxide (DMSO, Carl Roth, Germany) was added to each well and the plates were placed on the shaker for 5 min. Each investigated coating was tested in octuplicate once. The absorbance of each well was measured at 620 nm in a microplate reader (Multiskan FC Microplate Photometer, Thermo Fisher Scientific, China), and the percentage of cell survival was calculated. Finally, the dose–response curves were plotted to allow the calculation of 50% cytotoxicity concentrations (CC_50_) that cause lysis and death in 50% of cells 50. The principal scheme of the investigation is sketched in Figure 1c.

#### 2.4.3 Virucidal Efficacy Testing—Virus Treatment, and Virus Quantification

Suspensions of IBV and BoHV-1 in a volume of 0.01 mL were placed on 6 well tissue culture test plates and covered with the investigated different Cu content containing coatings or PVB-coated glass plate for control and incubated for 1 h at room temperature (Figure 1d). After incubation, the liquids were diluted 1:50 with medium containing 2% FBS and were used for the virucidal quantification (the determination of virus titer) and viral nucleic acid quantification employing real-time PCR. The virucidal efficacy of the investigated coatings that resulted in a change of viral load was evaluated by determining and comparing the TCID_50_ before (virus control) and after exposure on the surfaces of the investigated coatings (affected viruses). Cytopathogenic effect in sensitive cells was microscopically assessed after 72–120 h of virus incubation. The principal scheme of the investigation is sketched in Figure 1d. The TCID_50_ viral titers of IBV and BoHV-1 were calculated by the Spearman–Karber method.^51^

#### 2.4.4 Viral Nucleic Acid Quantification

##### 2.4.4.1 IBV Real-Time Taqman Reverse Transcription Real-Time Polymerase Chain Reaction

Real-time TaqMan reverse transcription polymerase chain reaction (RT-PCR) was used to compare the amounts of IBV RNA before (control) and after incubation with the investigated coatings. The RNA used in the real-time Taqman RT-PCR was extracted employing TRIzol Reagent (Thermo Fisher Scientific, USA) according to the manufacturer’s instructions. Real-time RT-PCR was performed as described by Meir.^52^ Briefly, a conserved region of 336 base pairs located at nucleotide position 741-1077 of the H120 strain N gene sequence (GenBank accession no. AM260960) was used to design primers and probe for the real-time RT-PCR assay. A forward primer (IBV-f), a reverse primer (IBV-r), and a TaqMan probe (IBV-p) were used to amplify and detect the 130-bp fragment. Details are provided in Table S1. Both the primers and probe were synthesized by Applied Biosystems, UK. PCR amplifications were carried out in a volume of 25.0 μL containing 12.5 μL of 2 × RT-PCR buffer mix (AgPath OneStep RT-PCR kit, Applied Biosystems), 1 μL of 25 × RT-PCR enzyme mix (Applied Biosystems), primers to a final concentration of 400 nM, probe to a final concentration of 120 nM, 2 μL of RNA template, and nuclease-free water. The reaction was carried out in StepOne Plus real-time PCR system (Applied Biosystems) at 45 °C for 10 min, 95 °C for 10 min, and 40 cycles of 95 °C for 15 s and 60 °C for 45 s. Amplification graphs were recorded and analyzed, and the threshold cycle (Ct) was determined with Mastercycler RealPlex2 (Eppendorf, Germany).^53^ The principal scheme of the investigation is sketched in Figure 1d.

##### 2.4.4.2 BoHV-1 Real-Time TaqMan PCR

BoHV-1 real-time TaqMan PCR was used to compare the amount of BoHV-1 DNA before (control) and after incubation with the investigated coatings. DNA isolation was carried out using a Genomic DNA purification kit (Thermo Fisher Scientific, USA, K0721). A protocol was used as described by Lelešius et al.^50^

BoHV-1 forward (BoHV-1-f) and reverse primers (BoHV-1-r) and probe (BoHV-1-p) for quantitative real-time PCR (TaqMan) were designed with Primer Express software (version 1.0; Applied Biosystems, USA) to amplify sequences (product size 97 bp) within the open reading frames of the glycoprotein B genes of BoHV-1.^54^ Oligonucleotide primers and MGB-labeled probes were synthesized by Invitrogen (USA) and are detailed in Table S2. Amplifications were performed using a TaqMan Universal master mix II (catalogue no. 4440038, Applied Biosystems, USA). Briefly, real-time PCR amplifications were carried out in a volume of 25.0 μL containing 12.5 μL of master mix, BoHV-1-f and BoHV-1-r (final concentration 240 nM each), BoHV-1-p (final concentration 160 nM), and 10.9 μL of the DNA template. The real-time PCR conditions for the reactions were set as follows: 2 min at 50 °C, 10 min at 95 °C, and then 40 cycles consisting of a denaturation step at 95 °C for 15 s and an annealing-elongation step at 60 °C for 1 min. Amplification plots were recorded and analyzed, and Ct was determined using a Mastercycler (Eppendorf). The real-time PCR was repeated four times, and Ct values were recorded.

### 2.5 Cu Release into the Medium

An identical set of samples was immersed in a 2 mL of DMEM cell maintenance medium for 1 h at room temperature. The concentrations of Cu released from the coatings into the medium were determined using a double-beam atomic absorption spectrometer AAnalyst 400 (PerkinElmer, USA). Quantitative analysis of the Cu amount was carried out using the calibration curve, which was determined by measuring reference solutions. Afterward, the optical density of the test solutions was measured, and the concentration of the test element in the solution was determined from the calibration curve.

### 2.6 Statistical Analysis

The results were analyzed with Origin Lab 2023, and the mean comparative studies of NP sizes, TCID_50_, and Ct estimation were done by two-way analysis of variance with the quantitative data expressed as the mean ± standard deviation. A statistical significance was considered at *p < 0.05. Linear regression and the square of the Pearson product–moment correlation coefficient (*R*^2^) were used for the evaluation of the correlation between Cu content and cytotoxicity, virus titer, and PCR Ct values.

## 3 Results

### 3.1 Cu NPs

**Figure 2:**
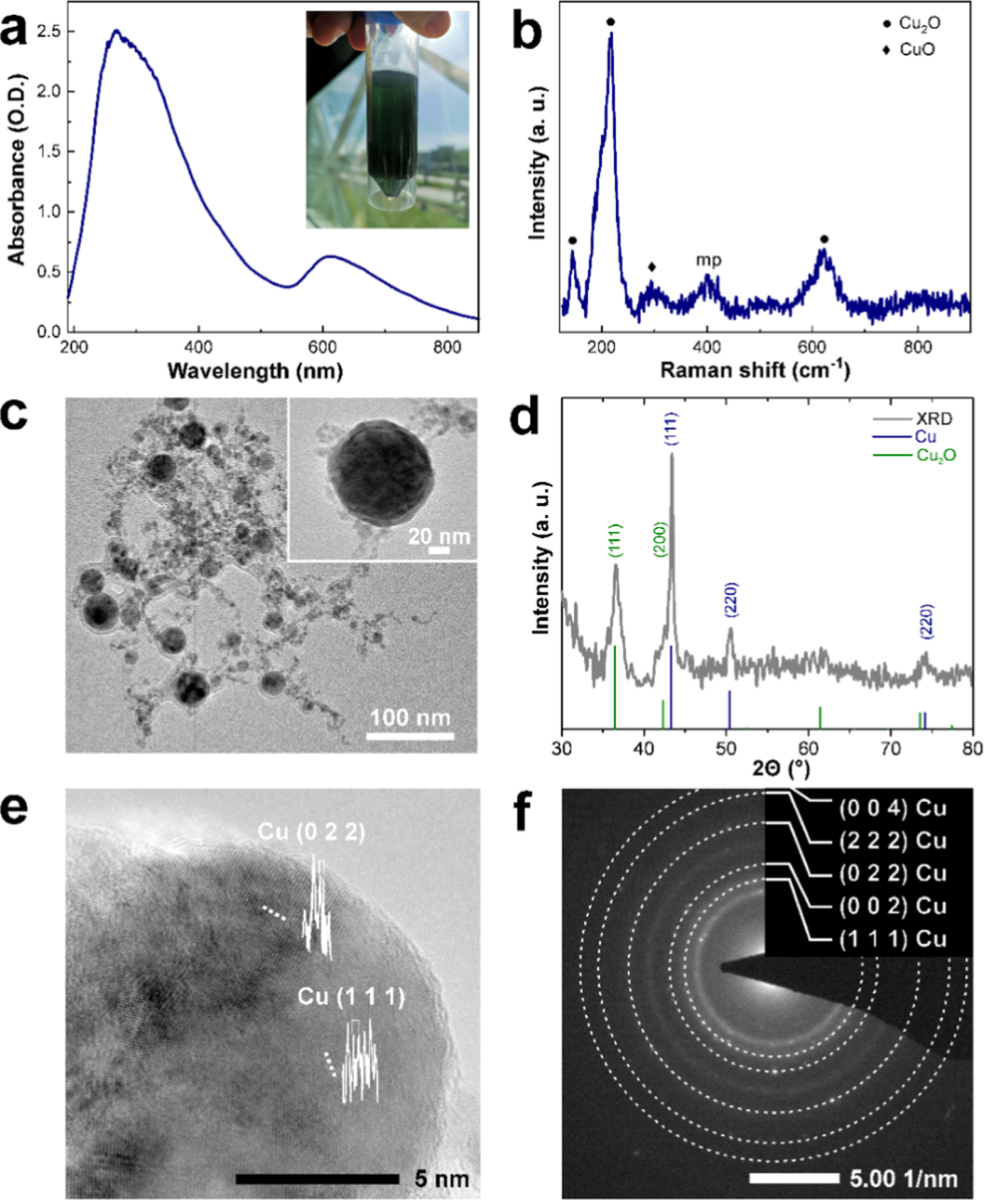
Analysis of the Cu colloid synthesized using sodium citrate surfactant. (a) UV–vis–NIR absorbance (optical density, O.D.) spectra of the concentrated colloid. The inset shows a camera image of the concentrated Cu colloid. (b) Raman scattering spectra of the CuNPs with the identified phases addressed in Table S4, “mp”—multiphoton, “a.u.”—arbitrary units. (c) TEM micrograph of the Cu NPs. (d) XRD of the Cu NPs with the indicated *hkl* indexes for Cu and Cu2O. (e) HRTEM micrograph of Cu NPs with facets addressed to metallic Cu. (f) SAED analysis of the Cu NPs with identified *hkl* indexes and metallic copper phases.

After the femtosecond laser ablation of the Cu target in liquid, the translucent color of the solvent changed into pale green (Figure 2a, inset, Figure S2a,b insets), indicating the formation of the Cu NP colloid. The absorption peak visible in the optical density spectra at ca. 620 nm is related to the LSPR of Cu NPs (Figure 2a). The bigger amplitude peak at ca. 300 nm is addressed to the interband transition of Cu.^20^ Without the addition of surfactant, Cu colloid NPs tended to oxidize and change color into pale yellow days after the process (see Figure S2b). Therefore, within this work, a separate study was carried out where the colloid optical properties were inspected over time after synthesis in pure water and using different sodium citrate concentrations from 0.002 to 2 mmol. Some red-shift in the extinction peak (Figure S3), as well as the decrease in the amplitude, was observed for all samples, whereas the 0.02 mmol sample was found to be the most stable and the only one that preserved greenish color over a period longer than 1 year. Andal and Buvaneswari^26^ reported similar results in the color change, where the presence of Cu_2_O was confirmed by the XRD method. In this work, the XRD diffractogram for the Cu NPs synthesized using surfactant indicated similar peaks but of different intensities, supporting the domination of metal Cu in a mixture with Cu_2_O (Figure 2d). On the contrary, others claimed to have green appearing laser synthesized CuO,^55^ but Cu and Cu oxides are not stable and tend to change within hours, as reported for chemically synthesized CuO that are prone to transform into Cu_2_O.^56^ The XPS study of nanocomposite Cu containing amorphous carbon films and their aging in aqueous media indicated the film color change from metallic to green which appeared to be the change of initial metallic copper and copper(I) oxide phases in pristine films into copper(II) oxide and copper(II) hydroxide in exposed films.^37^ Raman scattering measurements were carried out to confirm the material composition, as the Raman activity is related to the function of the space group symmetry of a crystalline solid.^20^ The spectra obtained with peaks characteristic for CuO and Cu_2_O^57^ are shown in Figure 2b and detailed in Table S3. The presence of oxygen in Cu NPs was also confirmed by the EDS mapping of the drop-cast Cu NP colloid on the crystalline silicon surface (Figure S4). High-resolution TEM images also indicated the presence of a metallic Cu phase (Figure 2e), where Cu(111) 0.239 nm and Cu(022) 0.147 nm atomic plane distances were identified. While for the month-aged sample synthesized in pure water (Figure S2d) and the year-aged sample that was synthesized using surfactant (Figure S2f), Cu/CuO and Cu/Cu_2_O phases were identified, respectively. The SAED patterns obtained for the fresh sample depicted in (Figure 2f) were addressed to Cu(111), (002), (022), (222), and (004), which confirms the presence of metallic Cu. After aging, CuO (Figure S2c) and Cu_2_O (Figure S2e) phases emerged. More detailed results analysis of the aged Cu NP samples synthesized in pure water and using surfactant are provided in the Supporting Information.

The size distribution of the Cu NPs obtained from the SEM micrograph (Figure S5a) is depicted in Figure S5b. The mean diameter of the NPs was 32 ± 14 nm. The shape of the Cu NPs can be assessed better in the TEM micrograph (Figure 2c), and it resembles spheres, which is expected for laser-ablated NPs.

The preliminary expenses analysis based on the consumed chemicals necessary to produce 100 mL of the colloid solution suggested that the photophysical synthesis cost was at least twice lower than wet-chemical synthesis, as explained in more detail in the Supporting Information. Such volume is further concentrated and is consumed in the typical spray-coating process resulting in a batch of samples for virucidal testing.

### 3.2 Coatings Containing Copper NPs

The Cu NP ink was spray-coated on PVB-coated glass substrates to monitor the UV transmittance and, therefore, qualitatively control the effective thickness of the deposited coating. The Cu content in the coatings was evaluated quantitatively by energy-dispersive X-ray spectroscopy (EDS). The EDS spectra are depicted in Figure S6, where one can see the emergence of the Cu-related L_α_ peak with increasing spray coating duration. This correlates with an increase in UV extinction. A complete list of detected elements and their concentrations, including the substrate, is provided in Table S4. The normalized elemental composition of the coating, obtained after subtracting the elements attributed to the glass substrate and leaving only Cu, which is in the form of NPs, and C, which is mainly related to the PVB coating, is presented in Table 1.

**Table 1:** Normalized Elemental Coating Composition in Weight Percent (wt %) and Atomic Percent (at %) Together with Released Cu Concentrations in Cell Medium after 1 h Immersion^a^

sample no.	element concentration			released Cu mg/L
		C	Cu	
PVB + CuO 10%	at %	97.1	2.9	0.377
	wt %	86.3	13.7	
PVB + CuO 15%	at %	92.8	7.2	0.673
	wt %	71.0	29.0	
PVB + CuO 25%	at %	88.8	11.2	1.972
	wt %	59.9	40.1	

a The full coating composition is provided in Table S4.

**Figure 3:**
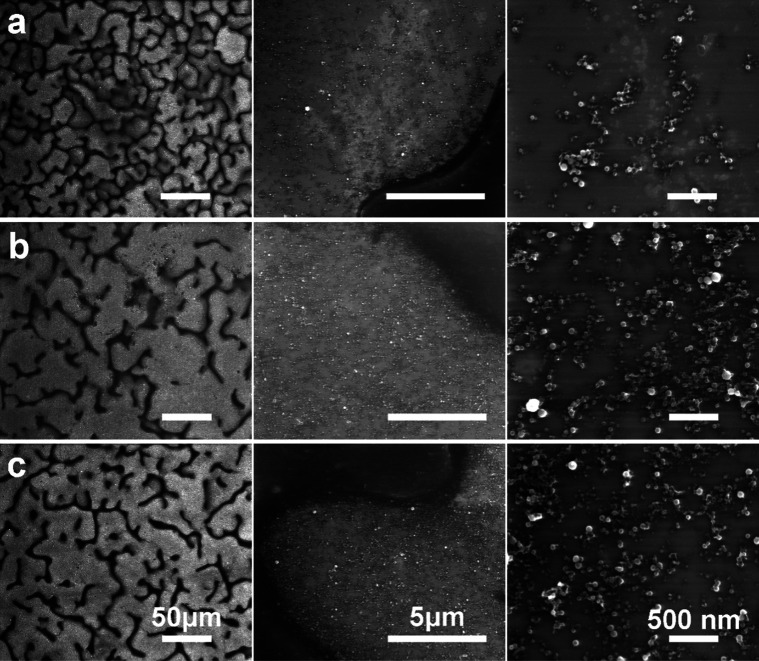
SEM micrographs of PVB coatings with different Cu NP content, (a) PVB + CuO 10%, (b) PVB + CuO 15%, and (c) PVB + CuO 25% at characteristic magnifications of 1 k×, 20 k×, 100 k× summarized in first, second, and third columns, respectively, with the scale bars of identified length.

Differences in surface morphology are summarized in Figure 3. PVB appears as a random mesh that stems from the droplets due to the chosen spray coating method, as seen on the micrometer range scale magnification (Figure 3, first column). At the nanometer scale level magnification, Cu NPs are seen either embedded or found in the valleys between PVB mesh (Figure 3, third column).

Despite the Cu NP loading, the spray-coated PVB surfaces can be cleaned by regaining the materials. Copper can be chemically etched in acidic or alkaline solutions and regained by precipitation, chemical reduction, or electrochemical deposition. The PVB matrix can be dissolved in the organic solvents and reused as described in more detail in the Supporting Information. These findings suggest that the materials used for the virucidal coatings are sustainable, as they are recyclable and reusable. The mechanical resistance of the typical PVB Cu NP coating sample surface coating hardness test after applying specific downward forces is depicted in Figure S7. The scratches start showing when using a 2 ± 1 N force, and the area of scratches increases with increasing forces.

### 3.3 Cytotoxicity Control

Cytotoxicity control was performed to differentiate cytotoxic and noncytotoxic Cu concentrations for Vero and MDBK cells and choose the correct concentrations for further virus treatment, ensuring unbiased results. It was obtained that PVB + CuO 10%, PVB + CuO 15%, and PVB + CuO 25% coatings were cytotoxic for both Vero and MDBK cell lines and caused the death of 50% of cells under dilutions identified in Table 2. The leached copper content in the determined dilutions was taken from Table 1, where identical samples were immersed in 2 mL of medium. It was found that the cytotoxicity correlated linearly with the content of Cu in the coatings, while linear correlation with the Cu released in the medium was less expressed (Table 2). Virucidal assays were carried out with higher dilutions than identified in Table 2, ensuring that cytotoxicity from investigated films is not prevailing and is not influencing the virucidal assay, ensuring unequivocal results.

**Table 2:** Cytotoxicity of Copper NP Coatings for Vero and MDBK Cells and the Correlation with the Initial Cu Concentration in the Film at % and Cu Released in the Medium

cells	cytotoxicity, CC_50_ (dilution factor/concentration μg/mL)			*R*^2^^a^	
	Cu NP coatings				
	PVB + CuO10%	PVB + CuO15%	PVB + CuO25%	Cu in film	Cu released
vero	0.64/2.36	1.18/2.28	1.49/5.29	0.90	0.60
MDBK	0.60/2.51	1.03/2.61	1.40/5.63	0.93	0.64

a *R*^2^ represents the correlation between the Cu concentration in the film at % or released in the medium and determined cytotoxic dilutions.

### 3.4 Virucidal Efficacy

**Figure 4:**
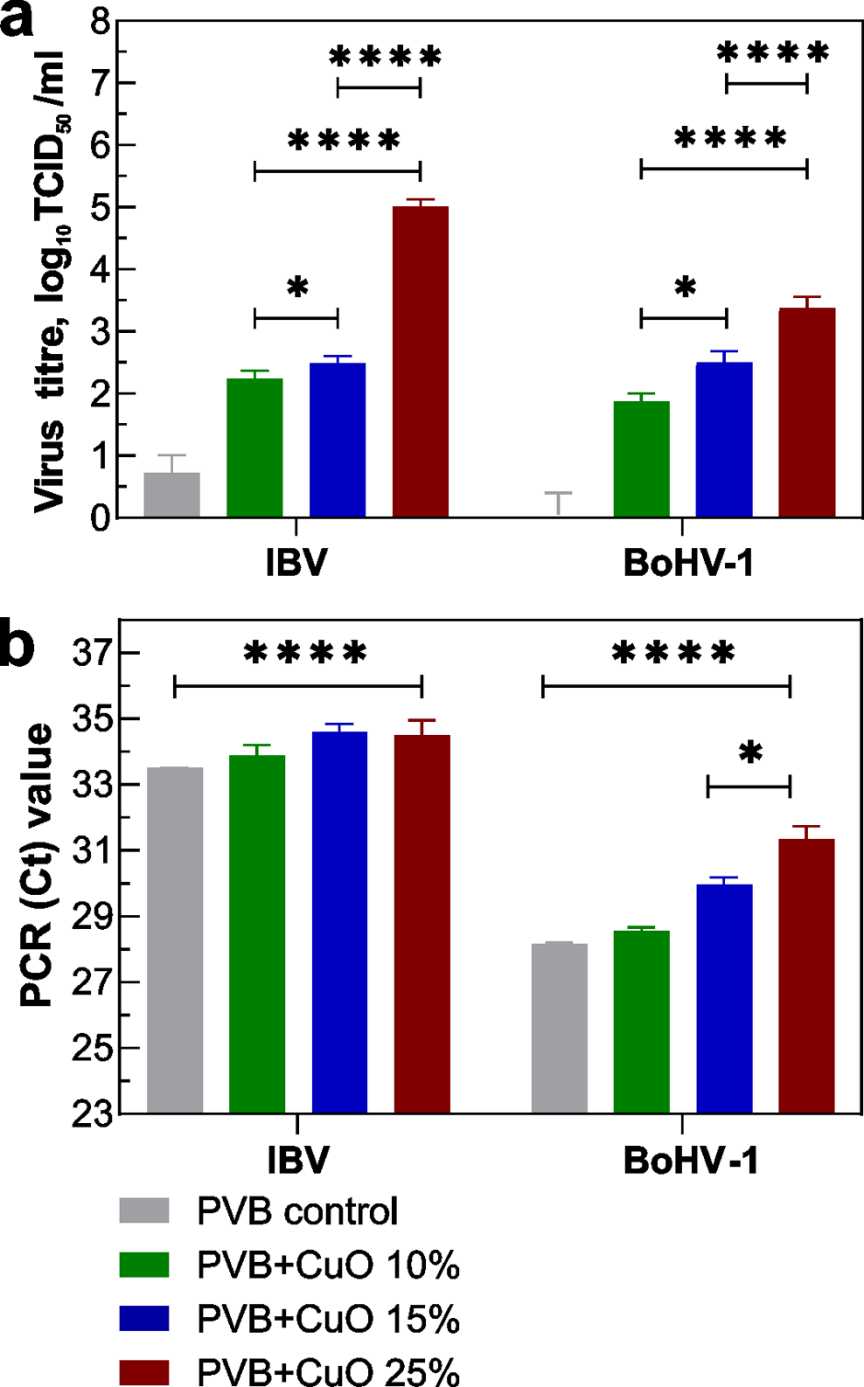
In vitro study of different PVB + CuO (10%, 15%, and 25%) coatings and control. (a) Virus titer reduction log10 of IBV, and BoHV-1 after 1 h of contact (*p = 0.0117 and ****p = < 0.0001) and PVB control. (b) Cycle threshold (Ct) values the virus control and coatings of the RT-PCR (*p = 0.0042 and ****p = < 0.0001).

The change in the number of two investigated type viruses after 1 h of contact incubations with different investigated coatings expressed as a decrease in log_10_ TCID_50_/ml, is depicted in Figure 4 and Tables S5 and S6. The Cu NP content in the coating directly influenced the virus activity. All concentrations significantly reduced IBV and BoHV-1 titer from 2.24 to 5.00 log_10_ TCID_50_/ml and 1.87 to 3.38 log_10_ TCID_50_/ml, respectively (Figure 4a). Samples of the highest Cu NP content coating (PVB + CuO 25%, or 11.2 at % of Cu) inactivated all coronaviruses (100%), while a 99.96% reduction was obtained for herpesviruses. Linear regression of the Cu content in the film with the TCID_50_ log_10_ virus reduction results in a coefficient of determination *R*^2^ = 0.80 and = 0.99, for IBV and BoHV-1, respectively, while calculating with the Cu content released in the medium *R*^2^ = 0.99 and = 0.94.

### 3.5 Real-Time PCR

The change in the number of nucleic acids of IBV and BoHV-1 after contact with the coating samples was evaluated with real-time PCR by determining the threshold Ct limit value increase from the PCR amplification plots. The cycle threshold values depicted in Figure 4b indicate that for IBV (Table S5), the PCR Ct values varied minimally from 33.5 to 34.6, while for BoHV-1 Figure 4b (Table S6), a significant Ct increase was registered from 28.6 to 31.4. Linear regression of the Cu content in the film with the Ct threshold value results in a coefficient of determination *R*^2^ = 0.65 and 1.00, for IBV and BoHV-1, respectively, while calculated with the Cu released in the medium *R*^2^ = 0.29 and 0.88.

## 4 Discussion

Laser ablation-based NP production offers a commercially viable alternative for green nanomaterial synthesis because only micrograms of the metal target were consumed to produce the colloid applied to produce the coatings. Taking into account the preliminary prices of the consumables for producing 100 mL of Cu NP colloid, the photophysical method is at least half as expensive as the traditional wet-chemistry synthesis approach. According to Jendrzej et al.,^13^ laser-based synthesis can be more economical if its productivity exceeds a certain breakeven value arising from the metal cost, which is orders of magnitude lower for copper than for gold, as per the original article.

The presence of the Cu_2_O phase in the photophysically synthesized NPs was confirmed by XRD, EDS, Raman, and SAED studies and indicated that the laser-ablated Cu colloids tend to oxidize. Still, they did not change their characteristic green color related to the LSPR of the Cu NPs in time when the 0.02 mmol concentration of sodium citrate surfactant was used. The presence of surfactant in water helped to prevent the fast aging of the Cu colloid that was observed by us and others.^20,44^ Smaller and higher surfactant concentrations also had a positive effect on colloid optical absorption spectra stability over time, which was effectively monitored as the amplitude of the plasmonic peak. For the smaller concentrations, the colloids were more stable than those made in ultrapure water. While for higher sodium citrate concentration, the barely detectable plasmonic peak amplitude indicated less efficient Cu NP synthesis from the very beginning. The aging process can also be related to the pH level, which can increase or slow the oxidation process,^58^ which can lead to a change in the NP’s virucidal properties.^59^ Exponentially increasing Cu release in the cell medium on the Cu content in the coating suggests that higher Cu NP loads in the coating have poorer binding with the surface. It might be related to longer deposition duration and, therefore, dissolving of the binding PVB agent. Similar nanocomposite coatings were investigated by Toledo et al.,^60^ where commercial Cu and CuO NPs were introduced into poly(methyl methacrylate) and polyepoxide matrixes and therefore resulted in an antiviral effect on human coronavirus HCoV-OC.^43^

The two-stage spray coating process enabled effective deposition and control of the Cu content on PVB-coated glass substrates and could be a more controlled alternative for dip coating and drop-casting, which were also used in similar studies.^31,33^ It was not investigated in more detail here, but the used setup can coat complex surfaces, for example, various touch surfaces^61^ and textiles^42^ because it is not limited by direct visibility like in fixed-source vacuum deposition systems but rather can access the surface of any pre-existing part utilizing a robotic arm or a hand-held applicator extending to multiple square meter area coverages at a small price.

Cu NP-containing coating antiviral studies were started from the cytotoxicity testing, aiming to assess their potentially destructive effects on different cell systems before evaluation of their virucidal effects. The testing on Vero and MDBK cell cultures revealed a similar impact on the viability of the cells. Cytotoxicity of copper nanomaterials depends on NP properties (size, shape, and surface) and environmental conditions (cells, medium composition, temperature, and pH), so the determination of a high range of cytotoxic concentrations is possible in different experiments that are not performed identically.^62^ The cytotoxicity of CuO NPs to various mammalian cell cultures was found to range from 9 to 25 μg/mL,^63^ and for bivalent copper ions (Cu^2+^), cytotoxicity in experiments is usually lower than that for copper oxide NPs. For example, a 12.7 μg/mL solution of Cu^2+^ was not cytotoxic to kidney cells.^64^ Our study showed that cytotoxicity has a direct linear correlation with the Cu content in the coatings and is also related to the Cu released in the medium. The Cu content release rate in 2 mL of medium volume might be different compared to the original experiment, where a 10 μL drop was spaced in a 100 μm thickness layer and therefore the initial Cu concentration gradients might have saturated faster. Similar data obtained from the MTT assay indicated a strong dose–response relationship concerning copper toxicity.^65^

Reduced cell viability at longer contact durations or bigger Cu concentrations can be related to the loss of the membrane barrier or cell surface microvilli that could be caused by oxidation stress.^66^ Studies with Cu NPs in canine kidney (MDCK) and liver (AML-12) cell cultures showed that the cell line viability decreased in a Cu concentration-dependent manner. The overproduction of reactive oxygen species (ROS) and mitochondrial membrane depolarization induced by CuO NPs led to disruption of the cell biological cycle in cell growth phases. Previous studies revealed distinct forms of cell death induced by CuO NPs in tested cell lines that exhibited a combination of apoptosis and autophagy.^67^ Cytotoxicity of copper was studied using copper sulfate compound in HeLa cells, and the mechanisms underlying copper-induced ROS production were observed. Exposure to copper sulfate affected HeLa cell viability in a dose-dependent way, subsequently increasing the subG1 and G2/M populations and corresponding decreasing the G1 population were observed. Copper sulfate also increased the levels of apoptosis, senescence, mitochondrial dysfunction, autophagy, ROS, and the expression of several stress proteins. In addition to HeLa cells, copper also induced cytotoxicity in human endometrial (HEC-1-A) and lung (A549) adenocarcinoma cells but not in normal human kidney (HEK293) or bronchial (Beas-2B) epithelial cells. Thus, the findings showed the differences in the functional roles of copper within cells.^68^ In our study, cytotoxicity CC_50_ for Vero cells, depending on the initial copper content in the coatings, ranged from 2.3 to 5.3 μg/mL, and for MDBK cells, from 2.5 to 5.6 μg/mL, respectively. The data we obtained showed that the cytotoxicity of Cu ions was higher than that indicated in the literature for copper-based NPs. However, according to other researchers, the cytotoxicity of ionic copper largely depends on the type of cell cultures used in the studies and ranges from 2.69 μg/mL (in gill cells) to 3.54 μg/mL (in hemocytes).^63^ In our research, we used NPs immobilized on the PVB coating surface. The greatest influence on cytotoxicity was caused by Cu ions entering the medium, which determined the cell cytotoxicity. Grillo et al.^69^ results indicate that a decrease in cellular mitochondrial activity under the influence of copper ions was observed at a concentration of ≥7.42 μg/mL and a plasma membrane integrity test showed a significant decrease in cell viability (almost 90%) at a concentration of 10.85 μg/mL. In addition, copper-induced DNA damage was detected in the concentration range of 5.67 to 7.42 μg/mL. Thus, considering the cell cultures used and the variable experimental conditions, the results of our copper cytotoxicity studies were similar to those obtained by other authors.

Cu NP coatings demonstrated expressed virucidal efficacy on both investigated RNA and DNA viruses. The test was carried out for 1 h due to some challenges in exploring definite results in a shorter time, as it did not show moderate or high virus reduction factor potential. This may be due to the relation of copper ion release in the aqueous medium, which is limited by the time-dependent diffusion effect. The reduction in the number of viruses after 1 h contact correlated with the atomic Cu concentration in the coatings, and it was more pronounced for the IBV than for the BoHV-1. The correlation with released Cu was more pronounced for IBV. This suggests that some differences in the Cu NP virucidal effect exist for the investigated RNA and DNA viruses. For the inactivation of IBV, contact with the coating might be the prevailing mechanism, while for BoHV-1, both the contact with the bonded NPs and the released Cu ion and Cu NPs might play a role. More detailed studies are necessary to support this hypothesis and understanding of the different mechanisms impacting the evolution over time. The absolute highest decrease in IBV biological activity of 100.0% was observed after contact with the PVB + CuO 25% sample throughout the entire scope of the study.

The real-time PCR results indicated different quantitative effects on the investigated viruses and their nucleic acids. The Ct threshold values followed the concentration trends only for the BoHV-1 virus, indicating the effect of Cu NP on DNA damage. It is important to note that PCR detects intact specific target sequences of viral nucleic acids of both live and inactivated viruses. Therefore, assessing the comparative percentage composition of nucleic acids of live and inactivated viruses in a sample by PCR alone is not possible. Previous studies showed that Cu NPs can have a direct degenerative effect on the biological structures of viruses, viral RNA, and short RNA fragments can be detected in postcontact titration and culture media of viruses.^32^ Our IBV real-time PCR confirmed the results of previous research and showed that Ct values are not the markers for infectivity of coronavirus.^70^ It was proved by both studies when RNA sequences were detected after inactivation of 100% coronaviruses. The comparison of the results of the quantification of viruses and their nucleic acids showed a higher decrease in virus infectivity than that in the degradation of nucleic acids. The data from our study with BoHV-1 also showed that the infectious titer of viruses (Figure 4a) after 1 h of contact with the surfaces decreased significantly faster than the DNA damage detected by PCR (Figure 4b). This could be explained by the fact that nucleic acids are significantly more resistant to external influences than viral proteins or envelope lipids.^71^

In our study, the high antiviral efficacy indicated by the virus titers can be addressed by the fact that the viruses were not resistant to Cu, which caused irreversible virus activity and possible RNA and DNA morphological and structural alterations. Multiple mechanisms having an internal effect on the virus were identified in literature including (i) the production of ROS through free copper ions (Cu^+^) released from the NP, leading to the denaturation of deoxyribonucleic acid (DNA)/RNA and damaged virion integrity;^8,38,72^ (ii) Cu^+^-induced virus inactivation by oxidizing lipids can inactivate viruses leading to the degradation of virus proteins through the generation of hydroxyl radicals;^8,73^ (iii) cytotoxicity caused by free radicals that interact with the components of the virus protein and generate hydroxyl radicals of transition metal hydroxyl radicals bound to the proteins;^8^ (iv) the inactivation of viral metalloproteins by replacing the respective metal with Cu;^74^ (v) disruption of the capsid integrity of the virus and destruction of DNA or RNA genomes by Cu binding and cross-linking between and within strands.^75−77^ The external antiviral properties of Cu NPs are related to (i) the “contact killing”^77^ and inactivation of the virus through membrane depolarization/or by some not-yet-explained mechanisms related to the dissolution of metal ions (Ag^+^, Cu^2+^, and Zn^2+^);^38,75^ (ii) surface-related catalytic activity of copper oxides (direct interaction with the surface),^75,77,78^ (iii) the interference with the virus capability to attach and enter target cells^79^ by rapidly damaging the virus surface proteins and membrane, breaking the envelope, or losing the virus capacity of self-containing folding upon itself.^11^

Since monovalent copper ions (Cu^+^) are more toxic than divalent (Cu^2+^), these ions might act as a catalytic cofactor for the formation of intracellular ROS. As a result of such exposure, the defragmentation of viral RNA occurs, and short RNA fragments can be detected by PCR.^32^ The external S proteins are very important for the protection of the genomic materials of coronaviruses in the external environment and are necessary for the first two stages of the viral biological cycle—“attachment” and “entry” into the host cell.^80^ Due to the sensitivity of external morphological structures, the inactivation of enveloped viruses is even faster compared to nonenveloped viruses.^8^ The Cu concentration is a key determinant of antimicrobial performance, with surfaces containing 55 to 70% active Cu effectively eliminating pathogenic microorganisms, including human immunodeficiency virus, within a very short contact time.^81^ Also, it has been demonstrated that solid copper oxide has been proven to effectively inactivate the influenza virus during contact with Cu_2_O, inhibiting the functional capacity of the HA protein (one of the main surface antigens) and destroying its biochemical structures.^31^ So, our research confirmed that the antiviral effect of Cu-based NPs is universal in different virus systems. In addition, the size of Cu NPs is another important criterion for virucidal efficiency, where smaller NPs were reported to have better activity.^82^

The study confirmed the feasibility and effectiveness of the low-cost, scalable functional coating synthesis and high-throughput spray coating deposition technology based on Cu/Cu oxide NPs originating via green photophysical synthesis. The femtosecond laser ablation in water-based Cu/Cu oxide NPs synthesis is an ecofriendly synthesis alternative that minimizes toxic byproducts, making it suitable for large-scale applications in healthcare and public high-touch surfaces. The main findings of the sodium citrate surfactant effect on copper colloid aging could apply to a wider range of applications in biomedicine, photonics, and catalysis, not limited to antimicrobial or virucidal coatings. These combined benefits and high virucidal efficacy confirmed against RNA and DNA-based viruses highlight the novelty and superiority of our method compared to those of existing Cu NP-based antiviral materials.

## 5 Conclusions

It was demonstrated that copper target ablation in water with 0.02 mmol sodium citrate employing a femtosecond laser can stabilize the Cu NPs and protect them from rapid aging. Optical extinction control of the concentrated Cu NP ink spray coating on PVB-coated glass allowed varying the copper content from 2.9 to 11.2 at. %. Raman, EDS, and XRD studies confirmed that 32 nm mean-size NPs are mixtures of mainly metallic copper and copper(I) oxide, Cu_2_O.

Studies of the virucidal activity of RNA-containing coronavirus IBV and DNA-containing herpesvirus BoHV-1 in cell cultures after 1 h contact with the investigated coatings indicated a definite Cu content-dependent negative effect on the biological activity of both model viruses leading to virus inactivation and viral nucleic acid degradation.

The absolute highest decrease in IBV biological activity of 100.0% was observed after contact with 11.2 atom % Cu content coating releasing up to 1.972 mg/L Cu and its compounds in the medium throughout the entire scope of the study.

The real-time PCR results indicated different quantitative effects on the investigated IBV and BoHV-1 and their nucleic acids. The Ct threshold values followed the concentration trend for BoHV-1, indicating the effect of the Cu NP on DNA damage.

## Data Availability

The data sets used or analyzed during the current study are available from the corresponding author upon reasonable request.

## Supporting Information Available

Brief description of obtaining the resulting overall colloid after centrifugation and solvent exchange, IBV real-time TaqMan reverse transcription PCR primers and probe, BoHV-1 real-time TaqMan reverse transcription PCR primers and probe, Cu NP Raman scattering peak positions and their assignment for space groups, brief description of the aging of Cu NPs showing UV–vis–NIR analysis, SAED analysis, and HRTEM micrographs, trisodium citrate concentration influence on the photophysically synthesized Cu colloid extinction spectra, EDS mapping analysis of the Cu NPs drop-cast on a crystalline silicon surface, NP size distribution analysis, EDS spectra of PVB spray-coated glass and PVB loaded with different Cu NP content, elemental composition of the PVB and PVB + CuO coatings on the glass substrates in atomic percent (at %), PVB with Cu NP samples’ coating surface hardness measurements using a sclerometer, IBV strain “Beaudette” virus biological activity evaluation after 1 h contact with different Cu content coatings, and BoHV-1 strain “4016” virus biological activity evaluation with different Cu content coatings (PDF)
