# Supplementary material for: Virucidal Efficacy of Laser-Generated Copper Nanoparticle Coatings against Model Coronavirus and Herpesvirus
Source: ACS Appl Mater Interfaces. 2025 Apr 22;17(18):26431–44. doi: 10.1021/acsami.5c03330 (PMC12067380; doi:10.1021/acsami.5c03330)
Supplement: Supplementary file 1 — am5c03330_si_001.pdf [file am5c03330_si_001.pdf]

Supporting Information

Virucidal Efficacy of Laser-Generated Copper Nanoparticle Coatings Against Model Coronavirus and Herpesvirus

Shahd Bakhet[1], Rasa Mardosaitė[1], Mohamed Ahmed Baba[1], Asta Tamulevičienė[1,2], Brigita Abakevičienė[1,2], Tomas Klinavičius[1], Kristupas Dagilis[2], Simas Račkauskas[1], Sigitas Tamulevičius[1,2], Raimundas Lelešius[3,4], Dainius Zienius[3,4], Algirdas Šalomskas[4], Krišjānis Šmits[5], Tomas Tamulevičius[1,2*]

[1]Institute of Materials Science of Kaunas University of Technology, K. Baršausko St. 59, LT-51423, Kaunas, Lithuania

[2]Department of Physics, Kaunas University of Technology, Studentų St. 50, LT-51368, Kaunas, Lithuania

[3]Department of Veterinary Pathobiology, Lithuanian University of Health Sciences, Tilžės St. 18, LT-47181 Kaunas, Lithuania

[4]Institute of Microbiology and Virology, Lithuanian University of Health Sciences, Tilžės St. 18, LT-47181 Kaunas, Lithuania

[5]Institute of Solid State Physics, University of Latvia, 8 Kengaraga St., LV-1063 Riga, Latvia

*Corresponding author: T. Tamulevičius, tomas.tamulevicius@ktu.lt, Tel: +370 (37) 313432, Institute of Materials Science of Kaunas University of Technology, K. Baršausko St. 59, LT-51423, Kaunas, Lithuania

Figure S1: Brief description of obtaining the resulting overall colloid after centrifugation and solvent exchange.
Table S1: The IBV real-time Taqman reverse transcription PCR primers and probe.
Table S2: The BoHV-1 real-time Taqman reverse transcription PCR primers and probe.
Table S3: Cu NP Raman scattering peak positions and their assignment for space groups
Figure S2: Brief description of the aging of Cu NPs showing UV-Vis-NIR analysis, SAED analysis, and HRTEM micrographs.
Figure S3: Trisodium citrate concentration influence on the photophysically synthesized Cu colloid extinction spectra.
Figure S4: EDS mapping analysis of the Cu NPs drop-cast on a crystalline silicon surface.
Figure S5: NP size distribution analysis.
Figure S6: EDS spectra of PVB spray-coated glass and PVB loaded with different Cu NP content.
Table S4: Elemental composition of the PVB and PVB+CuO coatings on the glass substrates in atomic percent (at.%).
Figure S7: PVB with Cu NP samples' coating surface hardness measurements using sclerometer.
Table S5: IBV strain "Beaudette" virus biological activity evaluation after 1-hour contact with different Cu content coatings.
Table S6: BoHV-1 strain "4016" virus biological activity evaluation with different Cu content coatings.

In total 4 tubes with 15 ml of initial colloidal solution were centrifuged for 20 min at 6000 x g (Lace 16, COLO, Slovenia), then the water was removed, and sediments were redispersed in 10 ml of isopropanol (**Figure S1**). The second centrifugation proceeded in the same way, only for the redispersion of sediment, only 5 ml of isopropanol was used to concentrate the final solution that was used for spray coating.

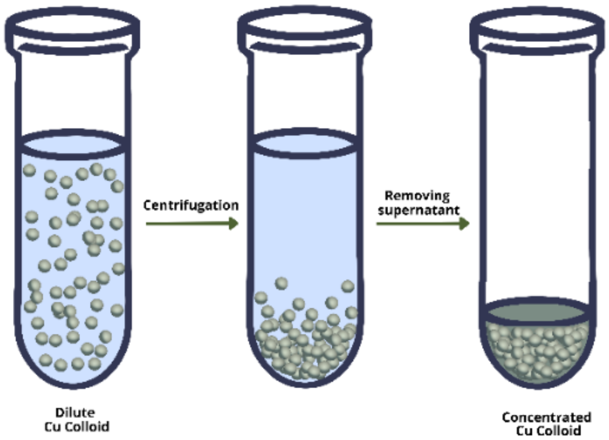

Figure S1 Concentration of the 15 ml of Cu colloid in aqeouos solution via centrifuging and exchange of solvent from water to isopropanol. The last two steps were repeated twice using 10 ml and 5 ml of isopropanol.

The forward primer (IBV-f), a reverse primer (IBV-r), and a TaqMan® probe (IBV-p) used in real-time Taqman reverse transcription polymerase chain reaction (PCR) are detailed in **Table S1**.

Table S1 The primers and probe for IBV real-time Taqman reverse transcription PCR

**Oligonucleotide**	**Sequence (5′-3′)**	**Nucleotide position**
IBV-f	ATGCTCAACCTTGTCCCTAGCA	811–832
IBV-r	TCAA-ACTGCGGATCATCACGT	921–941
IBV-p	FAM-TTGGAAGTAGAGTGACGCCCA AACTTCA-BHQ1	848–875

The forward (BoHV-1-f) and reverse primers (BoHV-1-r) as well as the probe (BoHV-1-p) for real-time TaqMan PCR are detailed in **Table S2**.

Table S2 The primers and probe for BoHV-1 real-time TaqMan PCR

**Oligonucleotide**	**Sequence (5′-3′)**	**Nucleotide position**
BoHV-1-f	TGTGGACCTAAACCTCACGGT	57499–57519
BoHV-1-r	GTAGTCGAGCAGACCCGTGTC	57595–57575
BoHV-1-p	AGGACCGCGAG TTCTTG CCGC	57525–57545

The Raman peaks of Cu NPs related to the function of the space group symmetry of Cu oxides are provided in **Table S3**.

Table S3 Cu NP Raman scattering peak positions and their assignment for space groups

No.	Peak position (cm^{-1})	Assignment
1	150	F_{1U} (Cu_2O) [1]
2	216	2 E_U (Cu_2O) [2]
3	296	Ag (CuO) [2]
4	405	Multiphoton process [1, 3]
5	620	F_{1U} (Cu_2O) [1]

The importance of the sodium citrate surfactant was investigated in the Cu NP colloid aging study. The UV-Vis-NIR spectra of freshly made Cu colloid in ultra-pure water were compared with the Cu colloid produced employing 0.02 mmol sodium citrate (NaCit, **Figure 2**) on the day of synthesis (**Figure S2 a**) and after one month of ageing in the dark (**Figure S2 b**). Additionally, Cu colloid synthesized employing sodium citrate and redispersed in isopropanol (as used for spraying) and stored in the dark for more than one year was included in the study. Their UV-Vis-NIR extinction spectra and TEM analysis results are depicted in **Figure S2 a, b**, and **Figure S2 c-f,** respectively. Cu colloid made in ultra-pure water after 1 month changed the initial green color (**Figure S2 a**) to pale yellow (**Figure S2 b**) while the colloid made using sodium citrate preserved a plasmonic peak.

Additional Cu colloid analysis was performed, tracking the changes in the absorbance in finer steps in time. The latter study was elaborated using different sodium citrate concentrations starting from pure water, 0.002 mmol NaCit, 0.02 mmol, 0.2 mmol, and 2 mmol. UV-Vis-NIR spectra were acquired every 1-5 days for approximately 3 weeks and around 1 year after the day of synthesis. The absorbance spectra were deconvoluted by subtracting the background signal under the LSPR peak at 600 nm (**Figure S3**). Examples of the full spectra are depicted in **Figure S2 a, b**. The initial results on day 1 suggest that the colloid is most effectively generated in pure water and 0.02-0.2 mmol samples, as the amplitude of the LSPR peaks is highest. The highest NaCit concentration resulted in the lowest magnitude LSPR peak, which deteriorated rapidly over time and was excluded from the study. The colloid in water indicated a decreasing amplitude over time, and no peak was found after a longer time. All colloids experienced a red-shift of their characteristic peak, but the sample with 0.02 mmol was the only one that preserved the absorbance peak after one year, and the changes of its peak intensity were least significant (**Figure S2**).

High-resolution TEM micrographs of Cu colloid made in ultra-pure water indicated the presence of metallic and oxidized copper in the CuO state as depicted in (**Figure S2 d**), where 0.203 nm and 0.237 nm atomic plane distances were identified, respectively. The SAED patterns of the Cu NPs depicted in (**Figure S2 c)** were addressed to Cu (111), which confirms the presence of metallic Cu. The presence of copper oxide in the films was confirmed by electron diffraction rings addressed to (111), (112), (-113), (-222),, and (131). For the second sample aged for one-year, high-resolution TEM images indicated the presence of metallic copper and copper II oxide as depicted in (**Figure S2 f**), where 0.204 nm and 0.237 nm atomic plane distances were identified. The SAED patterns of the Cu colloid are depicted in (**Figure. S2 e**) where rings are addressed to Cu (111), (222), which confirms the presence of a metallic Cu phase. The oxidized copper in the Cu_2O state in the colloids was confirmed by electron diffraction rings addressed to (111), (112), (022), and (222) planes.

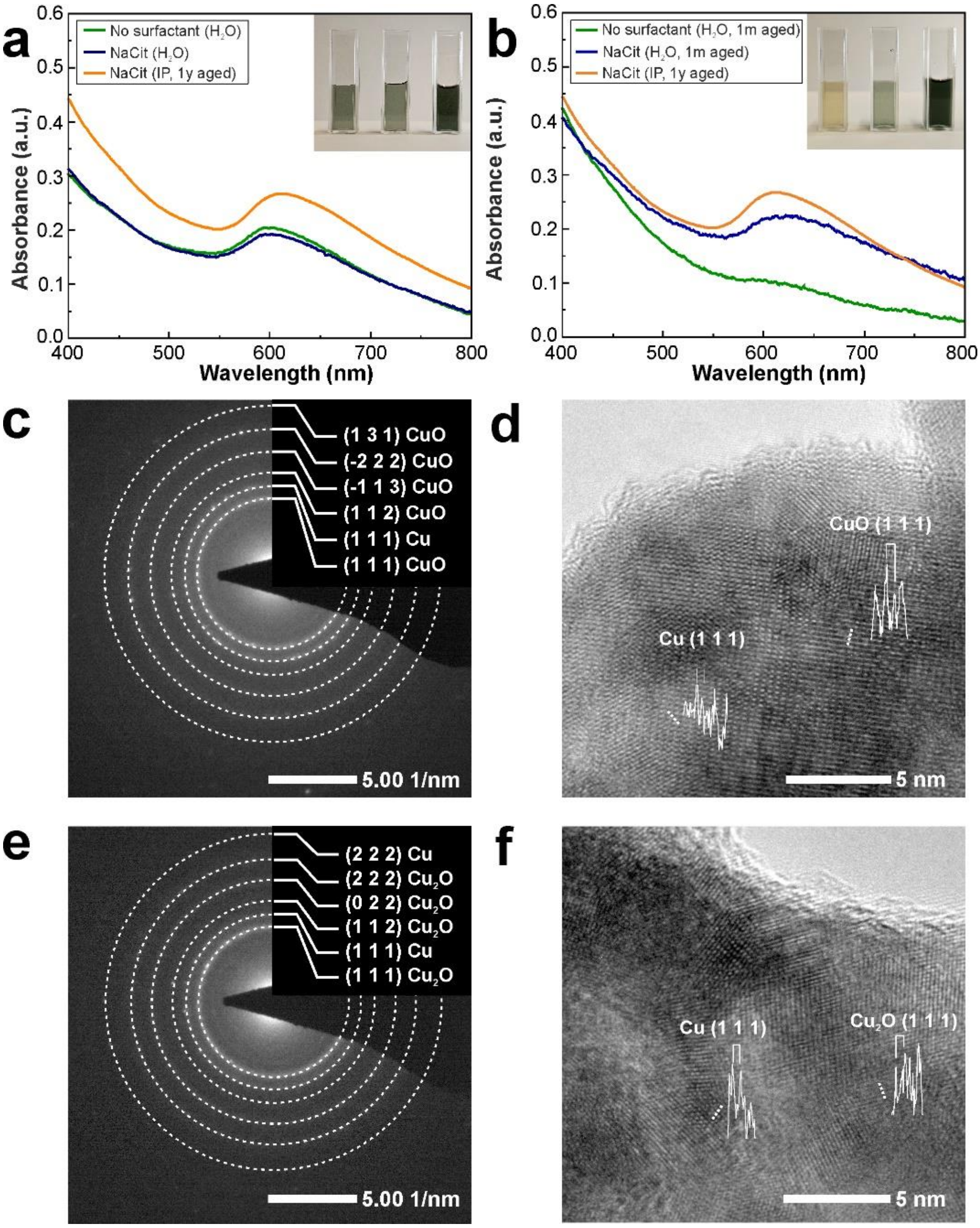

Figure S2 Aging of Cu NPs. UV-Vis-NIR analysis on the day of synthesis (a) and after one month (b) along with a concentrated one-year aged colloid produced using 0.02 mmol sodium citrate surfactant (NaCit). Insets depict camera images of the analyzed colloids from left to right: without surfactant in water, with sodium citrate in water, and with isopropanol (IP), in the cuvettes. HRTEM micrographs (d, f) and SAED analysis (c, e) of the Cu NPs made in ultra-pure water and aged for one month (c, d) and synthesized with sodium citrate surfactant and aged for one year (e, f). The facets in HRTEM and SAED micrographs were addressed to Cu, CuO, and CuO_2 phases with identified *hkl* indexes.

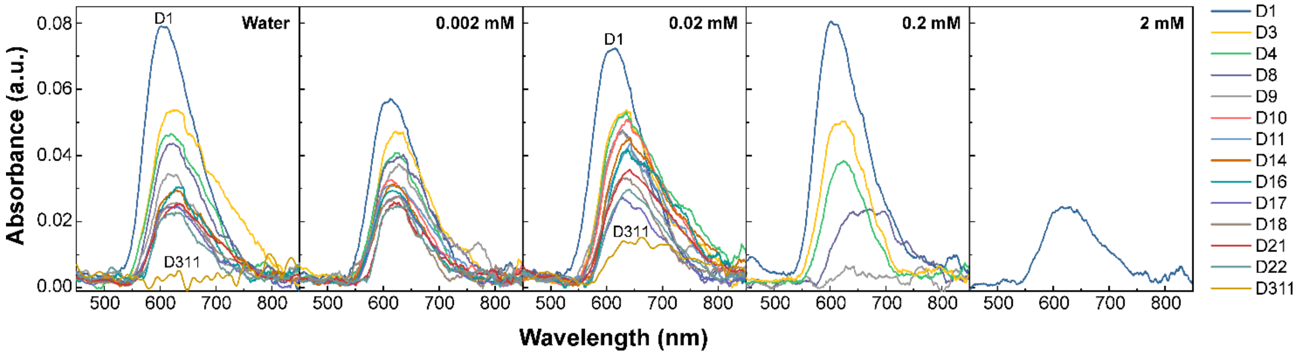

Figure S3. Subtracted background Cu colloid UV-Vis-NIR spectra after photophysical synthesis process utilizing different trisodium citrate concentration (pure water "Water", 0.002 mM, 0.02 mM, 0.2 mM, 2 mM). LSPR-related peak amplitude and position in the spectra acquired after different durations of time in days (D, indicated in the legend) manifest the stability of the Cu NP colloid.

Elemental EDS maps of drop-cast Cu NPs on a silicon substrate are depicted in **Figure S4**.

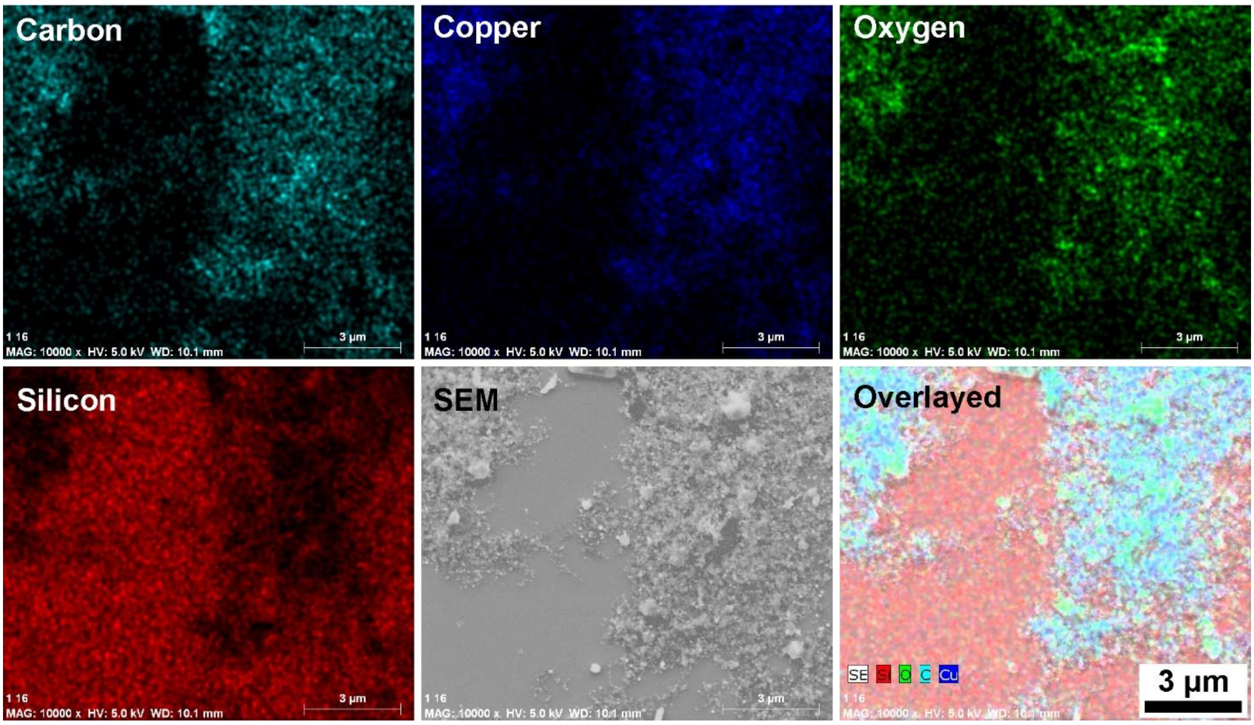

Figure S4 EDS mapping analysis of the Cu NPs drop-cast on a crystalline silicon surface. The detected chemical elements, namely carbon, copper, oxygen, and silicon, are indicated on the maps along with the SEM micrograph and overlaid elemental distribution. The measurements were obtained with a 5 kV accelerating voltage.

The SEM was used to define the size distribution of the Cu NPs which are depicted in **Figure S5.**

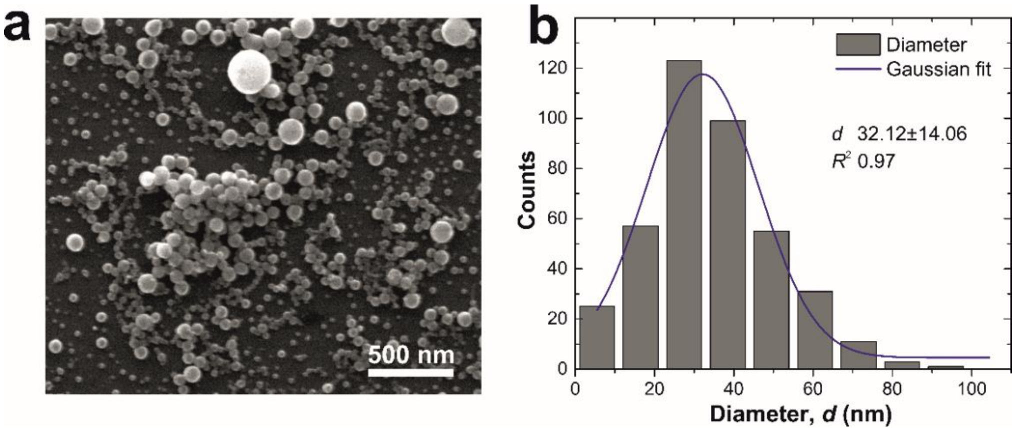

Figure S5 NP size distribution analysis. SEM micrograph of the Cu NPs drop-cast on a silicon substrate (a) and their size distribution (b).

The preliminary cost analysis of the laser-based photophysical and wet-chemistry synthesis of Cu NPs was conducted, which highlighted the economic advantage of the proposed synthesis method over the traditional one while maintaining the efficiency and sustainability of the produced NPs. To simplify the explorative price estimations, the consumption of energy, time, and labor costs were not taken into account, limiting the comparison of different syntheses to the consumption of chemicals. The laser-based synthesis requires only a metal target and a surfactant. Similarly, two precursors were used to chemically synthesize stable nanosized Cu NPs, as per a paper by Xiong *et al.* [4]. The photophysical method demonstrates at least a 2-fold cost reduction compared to the conventional chemical method. The main savings are related to the lower consumption of reducing/stabilizing agents and still minimal use of Cu precursor. Despite the energy footprint was not taken into account, laser-based synthesis is a cost-effective and environmentally friendly procedure because it does not require the use of fume hoods, heaters, and is also labor-effective as the process is automated and could be up-scaled. Particular research groups are focusing on cost-effective high-throughput photophysical synthesis issues, which we did not investigate in this work. For example, Khairani *et al.* [5], discuss pulsed laser ablation in water process limitations and demonstrate how it can be tweaked and upscaled by, for example, parallelizing and ablating with multiple beams or optimizing the individual beam scanning strategies, which can increase the synthesis yields from micrograms per hour to eventually grams per hour [5]. It has been demonstrated by Jendrzej *et al.* [6], that in the case of gold NPs, there exists a certain limit of 550 mg/h when industrial-level laser-based synthesis is more economical than chemical synthesis, where costs arising are limited by the metal costs, even taking into account the expensive hardware. In our case, copper prices are down to 4 orders of magnitude lower than gold, and therefore, it is even more economically viable.

EDS spectra of the PVB and PVB+CuO (10%, 15%, 25%) coatings are depicted in **Figure S6**.

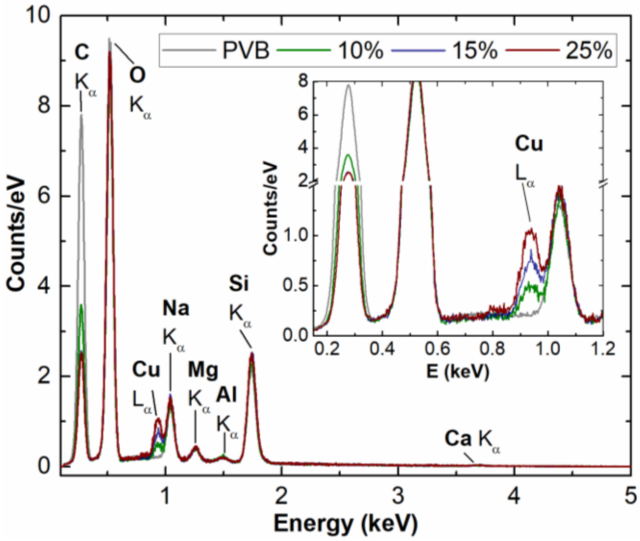

Figure S6 EDS spectra of PVB spray-coated glass and PVB loaded with different Cu NP content PVB+CuO 10%, PVB+CuO 15%, PVB+CuO 25% coatings. The measurements were obtained with a 5 kV accelerating voltage. The inset highlights the characteristic energy range for carbon and copper that are attributed to the coating. The characteristic X-ray peaks are noted with chemical elements along with the Siegbahn transition notation.

The full elemental composition of the PVB and PVB+CuO (10%, 15%, 25%) coatings of glass substrates obtained with EDS are provided in **Table S4**.

Table S4 Elemental composition of the PVB and PVB+CuO coatings on the glass substrates in atomic percent (at.%).

	Element	PVB	PVB+CuO 10%	PVB+CuO 15%	PVB+CuO 25%
**Coating**	Carbon (C)	45.33	28.62	21.25	20.34
	Copper (Cu)	-	0.86	1.64	2.57
**Substrate**	Oxygen (O)	42.22	52.89	57.20	57.28
	Sodium (Na)	1.50	2.15	2.53	2.50
	Magnesium (Mg)	0.39	0.47	0.52	0.62
	Aluminium (Al)	0.16	0.24	0.17	0.21
	Silicon (Si)	8.36	12.33	14.18	14.15
	Calcium (Ca)	2.04	2.44	2.44	2.33
		100.00	100.00	100.00	100.00

The investigated PVB coatings with Cu NPs could be treated as recyclable because it is possible to regain the PVB and Cu materials from the spray-coated surfaces. According to literature, the Cu NPs could be etched out using liquid chemical solutions for example, acidic ($FeCl_3$, HCL + H_2O_2) or alkaline solutions ($CuCl_2$ + NH_4OH) [7]. The Cu could be regained using precipitation (using Fe or Al), chemical reduction (ascorbic acid), or electrochemical deposition [8]. The polyvinyl butyral (PVB) spray-coated surfaces can be cleaned from the glass substrate using different methods, for example, using organic solvent, heat treatment, or mechanical scraping. The regained PVB solution can be reused as a glue for various materials, including glass, metals, ceramics, etc. [9]. The mechanical resistance of the spray-coated PVB with Cu NPs coating on a glass substrate was tested using a sclerometer 3092 (Elcometer) according to standard AS 3894.4 (EN 438-2, ISO 4586-2). The tip of the sclerometer was placed on the samples perpendicular to the surface, and the gauge was moved

across the surface to produce a linear scratch while applying a specific downward force of 1, 2, and 3 N which was applied through different springs. The gauge was then removed and the impact on the coating was examined under an optical microscope B-600MET (Optika, Italy) with a 3.2 MPix CMOS digital camera Optical Pro 3 (Optika, Italy). Pristine and mechanically impacted sample micrographs are depicted in **Figure S7**.

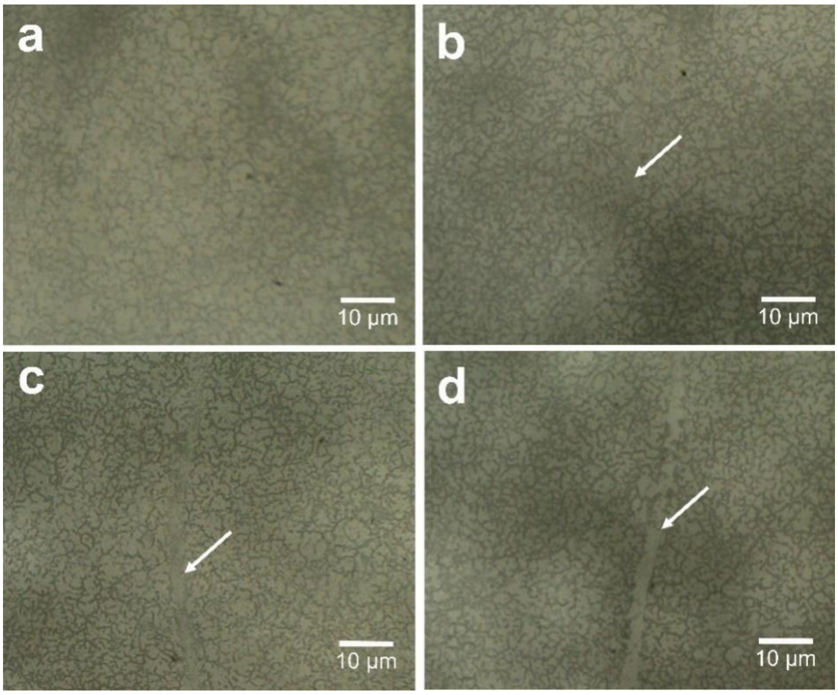

Figure S7 PVB with Cu NP samples' coating surface hardness measurements using sclerometer; (a) the control sample, (b) the sample scratched with 1 N force, (c) 2 N, (d) 3 N. Arrows indicate the position of the scratch.

Detailed results of IBV and BoHV-1 titres and real-time PCR are provided in **Table S5** and **Table S6**, respectively.

Table S5 IBV strain "Beaudette" virus biological activity evaluation after 1-hour contact with different Cu content coatings. 10 μl of the virus solution was used and contact duration with the investigated surface was 1 hour, temperature 20±2°C. Washed for 1 min with 490 μl DMEM (initial dilution – 1:50).

Sample	Viral titre, $\log_{10}$ $TCID_{50}$/ml, M±σ	Residual viruses, $TCID_{50}$ units/ml	A decrease in the number of viruses		PCR C_t value, M±σ
			$\log_{10}$ $TCID_{50}$ /ml	%	
IBV Control	5.00±0.13	100 000	0	0	33.5±0.02
PVB Control	4.40±0.29	25 100	0.60	74.89	33.0±0.50
PVB+CuO 10%	2.76±0.13	575	2.24	99.42	33.9±0.31
PVB+CuO 15%	2.51±0.12	323	2.49	99.68	34.6±0.24
PVB+CuO 25%	-	-	5.00	100.00	34.5±0.46

Table S6 BoHV-1 strain “4016” virus biological activity evaluation with different Cu content coatings. 10 µl of the virus solution was used and contact duration with the investigated surface was 1 hour, temperature 20±2°C. Washed for 1 min with 490 µl DMEM/F12 (initial dilution – 1:50).

Sample	Viral titre, $log_{10}TCID_{50}$/ml, M±σ	Residual viruses, $TCID_{50}$ units/ml	A decrease in the number of viruses		PCR C_t value, M±σ
			log_{10} $TCID_{50}$/ml	%	
BoHV-1 Control	7.20±0.0	15 848 931	0	0	28.17±0.05
PVB Control	7.15±0.23	14 125 375	0.05	10.87	27.80±0.33
PVB+CuO 10%	5.33±0.13	213 796	1.87	98.65	28.56±0.11
PVB+CuO 15%	4.70±0.19	50 118	2.50	99.70	29.97±0.21
PVB+CuO 25%	3.82±0.18	6 607	3.38	99.96	31.36±0.38

References

(1) Singhal, A.; Pai, M. R.; Rao, R.; Pillai, K. T.; Lieberwirth, I.; Tyagi, A. K. Copper(I) Oxide Nanocrystals - One Step Synthesis, Characterization, Formation Mechanism, and Photocatalytic Properties. *European Journal of Inorganic Chemistry* **2013**, (14), 2640-2651. DOI: 10.1002/ejic.201201382.

(2) Kwon, H.; Kim, J.; Ko, K.; Matthews, M. J.; Suh, J.; Kwon, H. J.; Yoo, J. H. Laser-induced digital oxidation for copper-based flexible photodetectors. *Applied Surface Science* **2021**, *540,* 7. DOI: 10.1016/j.apsusc.2020.148333.

(3) Meyer, B. K.; Polity, A.; Reppin, D.; Becker, M.; Hering, P.; Klar, P. J.; Sander, T.; Reindl, C.; Benz, J.; Eickhoff, M.; et al. Binary copper oxide semiconductors: From materials towards devices. *Physica Status Solidi B-Basic Solid State Physics* **2012**, *249* (8), 1487-1509. DOI: 10.1002/pssb.201248128.

(4) Xiong, J.; Wang, Y.; Xue, Q. J.; Wu, X. D. Synthesis of highly stable dispersions of nanosized copper particles using L-ascorbic acid. *Green Chemistry* **2011**, *13* (4), 900-904. DOI: 10.1039/c0gc00772b.

(5) Khairani, I. Y.; Mínguez-Vega, G.; Doñate-Buendía, C.; Gökce, B. Green nanoparticle synthesis at scale: a perspective on overcoming the limits of pulsed laser ablation in liquids for high-throughput production. *Physical Chemistry Chemical Physics* **2023**, *25* (29), 19380-19408. DOI: 10.1039/d3cp01214j.

(6) Jendrzej, S.; Gökce, B.; Epple, M.; Barcikowski, S. How Size Determines the Value of Gold: Economic Aspects of Wet Chemical and Laser-Based Metal Colloid Synthesis. *Chemphyschem* **2017**, *18* (9), 1012-1019. DOI: 10.1002/cphc.201601139.

(7) Cakir, O. Copper etching with cupric chloride and regeneration of waste etchant. *Journal Of Materials Processing Technology* **2006**, *175* (1-3), 63-68. DOI: 10.1016/j.jmatprotec.2005.04.024.

(8) Chen, J. P.; Lim, L. L. Recovery of precious metals by an electrochemical deposition method. *Chemosphere* **2005**, *60* (10), 1384-1392. DOI: 10.1016/j.chemosphere.2005.02.001.

(9) Królikowski, M.; Zach, P.; Kalestynski, M. Selection of Conditions in PVB Polymer Dissolution Process for Laminated Glass Recycling Applications. *Polymers* **2022**, *14* (23). DOI: 10.3390/polym14235119.
